# Estimating the Public Health Impact of Setting Targets at the European Level for the Reduction of Zoonotic *Salmonella* in Certain Poultry Populations

**DOI:** 10.3390/ijerph10104836

**Published:** 2013-10-11

**Authors:** Winy Messens, Luis Vivas-Alegre, Saghir Bashir, Giusi Amore, Pablo Romero-Barrios, Marta Hugas

**Affiliations:** 1 Unit on Biological Hazards (BIOHAZ), European Food Safety Authority (EFSA), Via Carlo Magno 1A, Parma 43126, Italy; E-Mails: luis.vivas-alegre@efsa.europa.eu (L.V.-A.); pablo.romerobarrios@efsa.europa.eu (P.R.-B.); marta.hugas@efsa.europa.eu (M.H.); 2 Unit on Scientific Assessment Support (SAS), European Food Safety Authority (EFSA), Via Carlo Magno 1A, Parma 43126, Italy; E-Mail: saghir.bashir@efsa.europa.eu; 3 Unit on Biological Monitoring (BIOMO), European Food Safety Authority (EFSA), Via Carlo Magno 1A, Parma 43126, Italy; E-Mail: giusi.amore@efsa.europa.eu

**Keywords:** *Salmonella*, target, poultry, breeding flock, laying hens, broilers, turkeys

## Abstract

In the European Union (EU), targets are being set for the reduction of certain zoonotic *Salmonella* serovars in different animal populations, including poultry populations, within the framework of Regulation (EC) No. 2160/2003 on the control of zoonoses. For a three-year transitional period, the EU targets were to cover only *Salmonella* Enteritidis and *S.* Typhimurium (and in addition *S.* Hadar, *S.* Infantis and *S.* Virchow for breeding flocks of *Gallus gallus*). Before the end of that transitional period, the revision of the EU targets was to be considered, including the potentially addition of other serovars with public health significance to the permanent EU targets. This review article aims at providing an overview of the assessments carried out by the Scientific Panel on Biological Hazards of the European Food Safety Authority in the field of setting targets for *Salmonella* in poultry populations (breeding flocks of *Gallus gallus*, laying flocks of *Gallus gallus*, broiler flocks of *Gallus gallus* and flocks of breeding and fattening turkeys) and their impact in subsequent changes in EU legislation.

## 1. Introduction

The presence of *Salmonella* in poultry populations is considered a risk factor for the presence of *Salmonella* in meat and eggs. To protect human health against *Salmonella* infections transmissible between animals and humans (zoonotic *Salmonella*), targets are set in the European Union for the reduction of certain *Salmonella* serovars in different animal populations, including poultry populations, within the framework of Regulation (EC) No. 2160/2003 on the control of zoonoses. Comparable data on the prevalence of *Salmonella* serovars in various poultry populations, *i.e.*, laying hens of *Gallus gallus* [1], broiler flocks of *Gallus gallus* [2], and turkey flocks [3], in EU Member States (MSs) have been collected through baseline surveys according to various Commission Decisions. For breeding hens of *Gallus gallus*, a baseline survey was not carried out. Details are provided in Table 1.

Within 18 months after EU-targets are agreed at the EU level, member states (MSs) have to prepare their National Control Programmes (NCPs), and submit them to the European Commission in order to get approval and possible co-financing. NCPs must include at least monitoring schemes and control measures as foreseen by EU legislation, but further sampling or specific methods can be added if considered appropriate based on the national epidemiological situation. The effectiveness of control measures applied is assessed through the achievement of the defined EU targets. 

For a transitional period of three years, the EU target was to cover only *Salmonella* Enteritidis and *S.* Typhimurium. In addition, for breeding flocks of *Gallus gallus* *S.* Hadar, *S.* Infantis and *S.* Virchow were considered, as these serovars were, together with the former, the five most frequently reported *Salmonella* serovars in human salmonellosis in the EU at the time. In this respect, it is worth noting that EFSA considered *Salmonella* monophasic strains with formula 1,4,[5],12:i:- as variants deriving from *S.* Typhimurium [4]. These monophasic *S*. Typhimurium strains have been shown to have similar virulence and antimicrobial resistance characteristics to other strains of *S*. Typhimurium and thus are considered to pose comparable public health risk to that of other epidemic *S*. Typhimurium strains.

Before the end of the transitional period, a review of the EU target should be carried out and other serovars with public health significance were considered for inclusion in a permanent EU target taking into account the criteria laid down in Annex III to Regulation (EC) No. 2160/2003. As stated in this Regulation (EC), the European Commission consulted the European Food Safety Authority (EFSA) before proposing EU targets.

In April 2008, the European Commission requested EFSA to assess the public health impact of the setting of a permanent target for the prevalence of *Salmonella* in flocks in certain poultry populations (*Gallus gallus*). This resulted in the publication of three EFSA scientific opinions from the Panel of Biological Hazards (BIOHAZ Panel). The first of the scientific opinions, published in April 2009, dealt with the impact of setting a new target for the reduction of the prevalence of *Salmonella* in breeding hen flocks of *Gallus gallus* [5]. A second and third scientific opinion, published respectively in April 2010 and July 2011, provided an estimation of the public health impact of setting new targets for the reduction of *Salmonella* in laying hen flocks [6] and broiler flocks [7]. In April 2012, following an additional request by the European Commission in June 2010, a fourth scientific opinion was published dealing with the assessment of the public health impact of setting new targets for the reduction of *Salmonella* in breeding and fattening turkey flocks [8].

For each of these scientific opinions, *ad hoc* working groups were established to draft the scientific opinions for consideration by the BIOHAZ Panel. In order to support the *ad hoc* working groups dealing with the last two scientific opinions (related to broilers and turkeys) EFSA commissioned modelling of the public health impact of target setting to external contractors (see Acknowledgements for details). With this article the authors aim to provide an overview of the risk assessments carried out by EFSA’s BIOHAZ Panel in the field of setting targets for *Salmonella* in poultry populations and the subsequent follow-up by the European Commission.

## 2. Experimental Section

### 2.1. Breeding Hens of *Gallus gallus*

EFSA was asked by the European Commission to assess the impact on the prevalence of *Salmonella* in flocks of broilers and laying hens if a new target for reduction of *Salmonella* was set in breeding hens. The new target would be 1% or less flocks remaining positive for all *Salmonella* serovars with public health significance, compared to the theoretical prevalence at the end of the transitional period (1% or less flocks remaining positive for five serovars), and to the real prevalence in 2007 that was reported by the MSs. The *Salmonella* serovars with public health significance were to be determined by EFSA taking into account the criteria laid down in Annex III to Regulation (EC) No. 2160/2003, *i.e.*, the reported frequency of serovars arising from human salmonellosis cases, the route of infection for these serovars, whether any serovar shows a rapid and recent ability to spread and to cause disease in humans and animals, and whether any serovar shows increased virulence for human infection.

A thorough literature review on the transmission of *Salmonella* in the poultry production chain, as well as an analysis of the *Salmonella* prevalence and serovar distribution correlation analysis between breeding and production flocks in the EU, were carried out [5]. The literature review considered the epidemiological aspects related to the different *Salmonella* transmission routes during primary production (*i.e.*, vertical, pseudo-vertical and horizontal transmission), and the relative importance of these different transmission routes. The *Salmonella* prevalence and serovar correlation analysis used EU data for the period 2004 to 2007 and data from Great Britain for the period 2000 to 2008. 

Finally, available risk assessment models were also considered for providing quantitative estimates on the impact of *Salmonella* prevalence in breeding flocks on its prevalence in production flocks [9,10]. Estimates on the expected prevalence of *Salmonella* in hatcheries and broilers depending on initial prevalence in breeding flocks were calculated based on the model developed by Nauta *et al.* [9].

**Table 1:** Timelines of setting *Salmonella* targets at the EU level in flocks of poultry populations and related regulatory instruments.

Zoonosis or zoonotic agent	Breeding flocks of *Gallus gallus*	Laying hens of *Gallus gallus*	Broilers of *Gallus gallus*	Breeding and fattening turkeys
**Baseline survey**				
Decision	NA ^a^	**Decision No. 2004/665/EC**	**Decision No. 2005/636/EC**	**Decision No. 2006/662/EC**
Technical specifications	NA ^a^	SANCO/34/2004 Rev.3	SANCO/1688/2005 Rev.1	SANCO/2083/2006
Time period	NA ^a^	Oct. 2004–Sept. 2005	Oct. 2005–Sept. 2006	Oct. 2006–Sept. 2007
Report part A published	NA ^a^	2007 [1]	2007 [2]	2008 [3]
**Transitional EU target and *Salmonella* NCP ^b^ in EU MSs**				
Regulation for EU target	**Reg. (EC) No. 1003/2005**	**Reg. (EC) No. 1168/2006**	**Reg. (EC) No. 646/2007**	**Reg. (EC) No. 584/2008**
Regulation for NCP	**Reg. (EC) No. 2160/2003**	**Reg. (EC) No. 2160/2003**	**Reg. (EC) No. 1177/2006**	**Reg. (EC) No. 2160/2003**
*Salmonella* target	≤1% *S.* Enteritidis, *S.* Hadar, *S.* Infantis, *S.* Typhimurium and/or *S.* Virchow	Annual reduction until ≤2% *S.* Enteritidis and/or *S.* Typhimurium	≤1% *S.* Enteritidis and/or *S.* Typhimurium	≤1% *S.* Enteritidis and/or *S.* Typhimurium
First year of harmonised monitoring and compulsory NCP	2007	2008	2009	2010
**EFSA’s risk assessment**				
Regulation	**Reg. (EC) No. 2160/2003**	**Reg. (EC) No. 2160/2003**	**Reg. (EC) No. 2160/2003**	**Reg. (EC) No. 2160/2003**
Mandate received (EFSA’s mandate number and question number)	7 Apr. 2008 (M-2008-0111; EFSA-Q-2010-291)	7 Apr. 2008 (M-2008-0111; EFSA-Q-2008-292)	7 Apr. 2008 (M-2008-0111; EFSA-Q-2008-293)	2 June 2010 (M-2010-0240; EFSA-Q-2010-00899)
Scientific opinion published	2009 [5]	2010 [6]	2011 [7]	2012 [8]
**Final EU target**				
Regulation	**Reg. (EC) No. 200/2010**	**Reg. (EC) No. 517/2011**	**Reg. (EC) No. 200/2012**	**Reg. (EC) No. 1190/2012**
*Salmonella* target	≤1% *S.* Enteritidis, *S.* Infantis, *S.* Hadar, *S.* Typhimurium ^c^ and/or *S.* Virchow	≤2% ^d^ *S.* Enteritidis and/or *S.* Typhimurium ^c^	≤1% *S.* Enteritidis and/or *S.* Typhimurium ^c^	≤1% *S.* Enteritidis and/or *S.* Typhimurium ^c^

^a^ NA = not applicable as for breeding hens a baseline survey was not carried out. Data was available from the European Summary Report from 2004 onwards; ^b^ NCP = National Control Programme; ^c^ Including monophasic *S.* Typhimurium with the antigenic formula 1,4,[5],12:i:-; ^d^ The annual targets are proportionate, depending on the prevalence in the preceding year, and the final EU target is defined as a maximum percentage of flocks remaining positive of 2%.

### 2.2. Laying Hens

EFSA was asked by the European Commission to assess the relative public health impact if a new target for reduction of *Salmonella* was set in laying hen flocks. The target would be 1% or less flocks remaining positive for all *Salmonella* serovars of public health significance, compared both to the theoretical prevalence at the end of the transitional period (2% or less flocks remaining positive for *S.* Enteritidis and/or *S.* Typhimurium), and to the real prevalence reported by the MSs in 2008. The *Salmonella* serovars with public health significance were to be determined taking into account the criteria laid down in Annex III to Regulation (EC) No. 2160/2003, as explained above for the first assessment.

The assessment [6] considered four different human exposure pathways to *Salmonella* from laying hens: internally contaminated table eggs, externally contaminated table eggs, egg products and meat from spent hens. 

To support the reply to this request and in particular for the table egg exposure pathway, EFSA built a quantitative risk assessment model of *S.* Enteritidis in shell eggs in Europe [11]. The model was based on the one employed by the Finish National Veterinary and Food Research Institute (EELA) in the quantitative risk assessment of *Salmonella* in egg production in Finland [12,13]. The initial structure of the EELA model was modified by using a continuous time variable, as described by Ranta *et al.* [13]. The two-stage EFSA model first estimated the average flock prevalence over a laying period in the production system. Following this, the model estimated the proportion of contaminated eggs for an infected laying hen flock. The combination of these two estimates results in the calculation of the expected number of eggs contaminated with *S.* Enteritidis per million of eggs. Suitable data for these analyses were obtained from two EU MSs.

### 2.3. Broilers

EFSA was asked by the European Commission to assess the relative public health impact if a new target for reduction of *Salmonella* was set in broilers. This target would be 1% or less flocks remaining positive for all *Salmonella* serovars of public health significance, compared to both the theoretical prevalence at the end of the transitional period (1% or less flocks remaining positive for *S.* Enteritidis and/or *S.* Typhimurium), and to the real prevalence reported by MSs in 2009. The *Salmonella* serovars of public health significance were to be determined by the EFSA as done in the previous assessments. 

A “Broiler-Target *Salmonella* Attribution Model” (BT-SAM), based on the so-called microbial subtyping attribution approach, was developed. The model considered the quantitative contribution and relevance to human salmonellosis of different *Salmonella* serovars found in broilers. The mathematical model was based on that developed by Hald *et al.* [14], in which the MSs were added as a third dimension. The basic principle in this model is to compare the serovar distribution observed in different animal-food sources with the serovar distribution found in humans. Detailed information on the methodology, mathematical principles, assumptions, data used, uncertainties and results of the model can be found in the external report provided by the contractor [15]. 

BT-SAM included 22 MSs, four animal-food sources (*i.e.*, broilers, laying hens, pigs, and turkeys) and 23 *Salmonella* serovars. The 23 serovars were selected based on their presence and importance in humans and in the animal-food sources. The monophasic *Salmonella* variant 1,4,[5],12:i:- was included in the model as *S*. Typhimurium. BT-SAM used (i) prevalence and serovar distribution data from the EU-wide baseline surveys (conducted in 2006–2007 for slaughter pigs [16], in 2006–2007 for fattening turkey flocks [3], in 2005–2006 for the prevalence data in broiler flocks [2], and in 2008 for the serovar data on broiler carcasses [17]), and from 2008 EU statutory monitoring data for laying hens; (ii) data on incidence and serovar distribution of reported cases of human salmonellosis in the EU in 2007 to 2009 (ECDC, TESSy Release 1 (6 July 2010) and 2 (28 October 2010 and updated on 5 May 2010; ECDC has no responsibility for the results and conclusions when disseminating the results of the work employing TESSy data supplied by ECDC), and (iii) food availability data, including amounts traded between MSs. MS-specific underreporting factors of human salmonellosis were also applied. The steps followed for selecting the data employed in the building of the BT-SAM model are described in detail in the scientific opinion [7]. In total seven scenarios were explored, where *Salmonella* prevalence in broiler flocks was changed and the results compared to the results of the baseline BT-SAM model.

### 2.4. Breeding and Fattening Turkeys

EFSA was asked by the European Commission to assess (1) the impact of a reduction of the prevalence of *Salmonella* in breeding flocks of turkeys on the prevalence of *Salmonella* in flocks of fattening turkeys and (2) the relative public health impact if a new target for reduction of *Salmonella* was set in turkeys. As for *Gallus gallus*, the target would be 1% or less of flocks remaining positive for all *Salmonella* serotypes with public health significance, compared to both the theoretical prevalence at the end of the transitional period (1% or less flocks remaining positive for *S.* Enteritidis and/or *S.* Typhimurium), and to the real prevalence reported by the MSs in 2010. As for broilers, the *Salmonella* serovars with public health significance would be determined by the EFSA as done in the former assessments.

In a similar way as for estimating the impact of target setting in broilers, a “Turkey-Target *Salmonella* Attribution Model” (TT-SAM) was applied. Detailed information can be found in the external report provided by the contractor [18]. 

TT-SAM included 25 MSs, the same four animal-food sources and 23 serovars as the BT-SAM model. It employed: (i) prevalence and serovar distribution data from the 2010 EU statutory monitoring (turkeys, broilers and laying hens) and the EU-wide baseline survey conducted in 2006–2007 for slaughter pigs [16]; (ii) data on incidence and serovar distribution of reported cases of human salmonellosis in 2010 (ECDC, TESSy Release on 6 October 2011; ECDC has no responsibility for the results and conclusions when dissiminating results of the work employing TESSy data supplied by ECDC), and (iii) food availability data, including amounts traded between MSs. MS-specific underreporting factors of human salmonellosis were applied too. The steps followed for selecting the data used to build the TT-SAM model are described in the scientific opinion [8]. Seven different scenarios were explored, where overall or serovar-specific prevalences in turkey flocks were changed and the results compared to those of the baseline TT-SAM model (2010 data).

In order to assess the impact of a reduction of *Salmonella* prevalence in breeding flocks of turkeys on the prevalence in flocks of fattening turkeys, information available in the literature and monitoring results about the presence of *Salmonella* serovars in turkey flocks at different levels were taken into account. Monitoring data were both from the EU baseline survey of 2006–2007 [10] and from the 2010 harmonised monitoring.

## 3. Results and Discussion

### 3.1. Breeding Hens of *Gallus gallus*

Based on the literature review, *S.* Enteritidis and *S.* Typhimurium were considered to have the greatest potential for vertical and pseudo-vertical transmission from breeding hens (*Gallus gallus*) to their progeny in the broiler meat and egg layer chains. EU-control measures for these two serovars in breeding hens were expected to contribute to the control of *Salmonella* infections in production stock, and thus to reduce human health risks from poultry. The marginal benefits of additional EU-wide control for other serovars in breeders (including the currently regulated serovars *S.* Hadar, *S.* Infantis and *S.* Virchow) were relatively small based on the outcome of the literature review as they have less potential for vertical transmission in particular for laying hens, as well as minimal relevance in terms of contamination of table eggs. It has to be acknowledged that biosecurity measures normally applied to control *S.* Enteritidis and *S*. Typhimurium would also have a beneficial effect to control horizontal transmission of other serovars.

In the EU, harmonised monitoring and reporting of *Salmonella* occurrence in different poultry populations was largely incomplete before NCPs were made compulsory [5]. Consequently, there were insufficient data to quantify the impact of controlling *Salmonella* prevalence in breeders on the prevalence in production stock in the EU. Further, it was highlighted that the datasets employed in the correlation analysis lacked both biological (*i.e.*, microbial subtyping correlation) and mechanistic (*i.e.*, breeding to production chain) information. Thus, any interpretation of the results obtained by this analysis would be difficult to interpret. Despite those limitations, some of the correlation analysis performed found a certain degree of temporal correlation between serovar occurrence in breeding and in production lines, being this stronger for *S.* Enteritidis and *S.* Typhimurium. Available risk assessment models were restricted to two EU MSs, and referred to earlier situations in which different control measures were implemented [9,10]. 

**Table 2:** Results of modelled flock prevalence in hatcheries and broilers, depending on different initial input values of prevalence in parents. Based on the model from Nauta *et al.* [9].

Starting prevalence in parents (%)	Estimated prevalence in hatcheries (%)	Estimated prevalence in broilers (%)
32	65.6	76.6
16	32.8	39.2
8	16.4	20.6
4	8.2	11.2
2	4.1	6.6
1	2.1	4.2
0.5	1.0	3.1

Based on model estimates [9], there were indications that, for those serovars for which vertical transmission is possible, control of *Salmonella* prevalence to very low levels is necessary in order to achieve a low prevalence in production stock (Table 2). 

### 3.2. Laying Hens

The model that was developed by EFSA [11] suggested a linear relationship between the prevalence of laying hen flocks and the number of eggs contaminated with *S.* Enteritidis per million of eggs produced. The model considered *S.* Enteritidis as the only serovar and eggs produced in the EU. Based on the median estimates from the model, changing from the EU average flock prevalence reported in 2008 being 3.1% to a transitional EU target of 2% is expected to result in around one third reduction in the number of contaminated eggs produced. Changing the EU target from 2% to 1% of positive flocks would result in a further reduction of a similar order of magnitude in the number of contaminated eggs produced. Besides the fact that *S.* Enteritidis was the only serovar considered, a limitation of the model was that steps of the table egg production chain beyond the laying phase were not considered (such as the packaging centres, catering, retail, consumer phase) [11].

The diversion of eggs from flocks that tested *Salmonella* positive to the production of egg products subjected to heat treatment may lead to increased public health risks. An increase was noted in reported non-compliance for microbial food safety criteria for egg products in 2008 compared to the two previous years. This is because applied heat treatments may not completely eliminate the risk of *Salmonella.*

There were insufficient data to quantitatively evaluate the public health risk associated with consumption of fresh meat from spent hens. However, based on the available data, it was anticipated that the *Salmonella* prevalence in the meat from these flocks might be higher compared to meat from broiler flocks, in particular if sourced from *Salmonella-*positive laying hen flocks.

### 3.3. Broilers

The results of the BT-SAM model indicated that the estimated true number of human salmonellosis cases (*i.e.*, when accounting for underreporting) in the EU in the combined period 2007 to 2009 was 8.8 million (95% credibility interval (CI): 8.4–9.2). 

**Table 3:** Percentage (%) of human salmonellosis cases in EU attributable to the four main animal reservoirs included in the BT-SAM [7,15] and TT-SAM model [8,18].

	Percentage of human cases (%)					
	BT-SAM model			TT-SAM model		
	Mean ^a^	2.5% ^b^	97.5% ^b^	Mean ^a^	2.5% ^b^	97.5% ^b^
Pigs	28.2	26.9	29.6	56.8	48.2	65.8
Broilers	**2.4**	**1.8**	**3.4**	10.6	5.1	18.3
Laying hens	65.0	62.8	67.1	17.0	11.3	24.0
Turkeys	4.5	4.0	5.0	**2.6**	**1.2**	**5.2**

^a^ Average or ‘centre of gravity’ of the uncertainty distribution; ^b^ Percentiles representing the low and high values across the range estimated by the model.

The model estimated that 4.5%, 65.0%, 28.2% and 2.4% of human salmonellosis cases could be attributed to the turkey, laying hen (eggs), pig and broiler reservoirs, respectively (see Table 3). 

This model considered the prevalence of *Salmonella* in broiler flocks as per the 2005–2006 baseline survey [2]. Broilers were estimated to correspond to around 207,250 (95% CI: 156,240–301,085) true human cases in the three-year combined period (*i.e.*, 2007 to 2009) out of a total of 8.8 million estimated human salmonellosis true cases.

The estimated number of human salmonellosis cases by the serovars included in the model and originating from the broilers reservoir are presented in Table 4. Around half of the broiler-associated human salmonellosis cases were estimated to be caused by serovars other than the currently regulated serovars *S.* Enteritidis and *S.* Typhimurium. *S.* Enteritidis and *S.* Infantis constituted 42% and 23% of all broiler-associated cases respectively. *S.* Hadar, *S.* Typhimurium, *S.* Kentucky and *S.* Virchow constituted individually between 4% and 5% of all broiler-associated cases. Other serovars constituted less than 4% on an individual basis.

**Table 4:** Estimated number of human salmonellosis cases by the serovars included in the model and originating from the broiler (from BT-SAM model) [7,15] and the turkey reservoir (from TT-SAM) [8,18].

Broiler reservoir (BT-SAM model)			Turkey reservoir (TT-SAM model)		
Serovar	Mean	% of total	Serovar	Mean	% of total
***S.* Enteritidis**	**87,513**	**42.2%**	***S.* Enteritidis**	**29,770**	**22.0%**
*S.* Infantis	47,665	23.0%	*S.* Kentucky	22,970	17.0%
*S.* Hadar	10,094	4.8%	***S.* Typhimurium ^a^**	**20,010**	**14.8%**
***S.* Typhimurium ^a^**	**9,649**	**4.7%**	*S.* Newport	10,030	7.4%
*S.* Kentucky	9,097	4.4%	*S.* Virchow	9,110	6.7%
*S.* Virchow	8,843	4.3%	*S.* Saintpaul	8,439	6.2%
*S.* Java	7,408	3.6%	*S.* Infantis	7,274	5.4%
*S.* Brandenburg	6,586	3.2%	*S.* Hadar	6,820	5.0%
*S.* Montevideo	5,037	2.4%	*S.* Bredeney	4,924	3.6%
*S.* Agona	3,820	1.8%	*S.* Agona	2,923	2.2%
*S.* Livingstone	2,961	1.4%	*S.* Kottbus	2,907	2.2%
*S.* Mbandaka	2,084	1.0%	*S.* Derby	2,445	1.8%
*S.* Derby	1,350	0.7%	*S.* Mbandaka	2,046	1.5%
*S.* Anatum	1,271	0.6%	*S.* Senftenberg	1,437	1.1%
*S.* Kottbus	1,236	0.6%	*S.* Bovismorbificans	1,157	0.9%
*S.* Braenderup	893	0.4%	*S.* Heidelberg	1,095	0.8%
*S.* Newport	675	0.3%	*S.* Montevideo	850	0.6%
*S.* Bredeney	607	0.3%	*S.* London	317	0.2%
*S.* London	206	0.1%	*S.* Livingstone	307	0.2%
*S.* Saintpaul	156	0.1%	*S.* Anatum	143	0.1%
*S.* Heidelberg	99	0.05%	*S.* Brandenburg	112	0.1%
*S.* Bovismorbificans	0	0.0%	*S.* Rissen	39	0.0%
*S.* Rissen	0	0.0%	*S.* Braenderup	0	0.0%
**Total**	**207,250**	**100%**	**Total**	**135,100**	**100%**

^a^ Including monophasic *S.* Typhimurium with the antigenic formula 1,4,[5],12:i:-.

Seven scenarios were evaluated, in which the prevalence of one or a combination of *Salmonella* serovars were lowered to 1% or less. From these, the four key scenarios, *i.e.*, those specifically addressing the questions in the mandate from the Commission, will be further discussed (Table 5). Considering the prevalence of the 23 *Salmonella* serovars included in the BT-SAM model to be as reported by the MSs in 2009, an estimated reduction of 69% (95% CI: 62–76%) in the number of broiler-associated human salmonellosis cases compared to the situation in 2005–2006 (baseline survey) [2] was expected. Considering the prevalence of *S.* Enteritidis and *S.* Typhimurium as that reported by the MSs in 2009 (but keeping the prevalence for the other 21 serovars as per the 2005–2006 baseline survey in broiler flocks) resulted in an estimated reduction in the number of broiler-associated human salmonellosis cases of 26% compared to the situation in 2006. Considering that the current target of the EU control programme of *Salmonella* in broiler flocks would be met (Scenario 3, *i.e.*, the combined prevalence of *S.* Enteritidis and *S.* Typhimurium being 1% or less), and keeping the prevalence for the other 21 serovars as per the 2005–2006 baseline survey in broiler flocks, an estimated reduction in the number of broiler-associated human salmonellosis cases of 25% compared to the situation in 2006 was expected. Achieving the EU-wide target of a maximum of 1% of broiler flocks remaining positive for the 23 *Salmonella* serovars considered in the model would lead to an estimated reduction in the number of broiler-associated human salmonellosis cases of 93%, compared to the situation in 2006.

**Table 5:** Estimated reduction in percentage (%) of human salmonellosis cases in the EU originating from the broiler reservoir [7,15] and the turkey reservoir [8,18] when compared to the baseline model under the different scenarios.

% reduction of all broiler-associated cases ^a^				% reduction of all turkey-associated cases ^h^			
	Mean ^b^	2.5% ^c^	97.5% ^c^		Mean ^b^	2.5% ^c^	97.5% ^c^
Scenario 1 ^d^	69.0	62.2	75.4	Scenario 1 ^i^	0.4	0.1	1.3
Scenario 2 ^e^	26.3	18.5	39.7	Scenario 2 ^j^	83.2	79.0	87.4
Scenario 3 ^f^	25.4	18.9	37.7				
Scenario 4 ^g^	93.4	92.9	94.1				

^a^ The baseline model uses the broiler flock prevalences as obtained through the EU baseline survey in broiler flocks conducted in 2005–2006 [2]. The serovar distribution was obtained from the EU baseline survey in broiler carcasses in 2008 [17]; ^b^ Average or ‘centre of gravity’ of the uncertainty distribution; ^c^ Percentiles representing the low and high values across the range estimated by the model; ^d^ The prevalence of the 23 *Salmonella* serovars included is as reported by the MSs in 2009; ^e^ The prevalence of *S.* Enteritidis and *S.* Typhimurium is as reported by the MSs in 2009 (but keeping the prevalence for the other 21 serovars as per the 2005–2006 baseline survey in broiler flocks, [2]); ^f^ The combined prevalence of *S.* Enteritidis and *S.* Typhimurium = 1% (or less) and keeping the prevalence for the other 21 serovars as per the 2005–2006 baseline survey in broiler flocks [2]; ^g^ The combined prevalence of all serovars in the model = 1% (or less); ^h^ The baseline model applies the turkey flock prevalences and serovar distribution data from the 2010 EU statutory monitoring; ^i^ The combined prevalence of *S.* Enteritidis and *S.* Typhimurium = 1% (or less) and keeping the prevalence for the other 21 serovars as per the 2010 reporting from MSs in turkey flocks; ^j^ The combined prevalence of all serovars in the model = 1% (or less).

The EU statutory monitoring in the MSs is likely to have a lower sensitivity in detecting positive flocks than the conducted EU-wide baseline surveys based on differences in the sampling scheme. For this reason, the estimated reductions in number of human salmonellosis cases were considered to be overestimated at the EU-level. Furthermore, it should be noted that the individual MS’ contributions to the estimated reductions varied greatly. 

### 3.4. Breeding and Fattening Turkeys

The results of the TT-SAM model (see [8,18]) indicate that the true number of human salmonellosis cases (*i.e.*, accounting for underreporting) in the EU in 2010 was estimated to be 5.4 million (95% CI: 3.0–9.5). The model estimated that 2.6%, 17.0%, 56.8% and 10.6% of human salmonellosis cases could be attributed to the turkey, laying hen (eggs), pig and broiler reservoirs, respectively (see Table 3). Turkeys are estimated to correspond to around 135,100 (95% CI: 60,790–293,600) true number of human cases in 2010.

Compared to the BT-SAM model [7,15], the TT-SAM model attributed a relatively high proportion of human salmonellosis cases to the pig reservoir. Partly, this can be explained by the different prevalence and serovar distribution in both the food-animal and human data used (influenced by the years considered in both models). Furthermore, the human salmonellosis cases in the EU has continuously decreased the last years with a particular decrease in *S*. Enteritidis cases [8], as explained by management interventions in the breeding and laying hen and broiler populations. As a consequence, the relative importance of other serovars and their reservoirs becomes more important. Nevertheless, an increase in the absolute number of *S.* Typhimurium (typically attributed to the pig and cattle reservoirs) cases is also observed, and is partly related to the emergence of monophasic variants (1,4,[5],12:i:-).

The estimated number of human salmonellosis cases by the serovars included in the model and originating from the turkey reservoir are presented in Table 4. Approximately 63.2% of the turkey-associated human salmonellosis cases were caused by serovars other than the currently regulated serovars *S.* Enteritidis and *S.* Typhimurium. However, these two serovars were still among the most important ones causing human infections originating from turkeys, constituting 22.0% and 14.8% of all turkey-associated cases, respectively. *S.* Kentucky constituted 17.0% of all turkey-associated cases. *S.* Newport, *S.* Virchow and *S.* Saintpaul constituted individually between 6% and 8% of all turkey-associated cases. Other serovars constituted less than 6% on an individual basis.

Seven scenarios were evaluated, in which the prevalence of one or a combination of *Salmonella* serovars was lowered to 1% or less. Two key scenarios, addressing the questions in the mandate from the Commission, will be further discussed (Table 5). Achieving the current EU-wide transitional target in fattening turkey flocks in all MSs, was expected to result in a further 0.4% (95% CI: 0.1–1.3%) reduction in the number of turkey-associated human salmonellosis cases compared to 2010. This corresponds to only 594 (95% CI: 121–1,901) out of the 5.4 million estimated human salmonellosis true cases. Achieving the EU-wide target of a maximum 1% of fattening turkey flocks remaining positive for the 23 *Salmonella* serovars considered in the model in all MSs, was expected to result in a 83.2% (95% CI: 79.0–87.4%) reduction in the number of turkey-associated human salmonellosis cases. This is equivalent to a 2.0% reduction of all human cases compared to 2010 and to a reduction of around 112,300 (95% CI: 50,410–243,400) true number of human salmonellosis cases. It should be noted that the individual MS’ contributions to the estimated reductions vary greatly.

For breeding turkeys it was concluded that the most frequently isolated serovars from breeding and fattening turkey flocks in the baseline survey appear to be similar for some MSs. Analysing results from the 2010 monitoring, five serovars were found both in breeders and in fattening flocks, one was present only in breeders, whereas 16 were found in fattening flocks but not in breeding flocks of turkeys. Although no quantification is currently possible, vertical transmission and hatchery-acquired infection appear as most important sources for *Salmonella* infection in fattening turkeys. It was also concluded that controlling the infection in breeders is necessary, but not sufficient, to control *Salmonella* in flocks of fattening turkeys.

## 4. Conclusions

The EFSA quantitative assessments of the impact of *Salmonella* targets in poultry populations in the EU on production flocks (*i.e.*, when targets are set for breeding flocks) and on public health (*i.e.*, when targets are set for production flocks) have been largely dependent upon two key aspects: data availability and modelling capacity.

The assessment of the impact of setting a new target in breeding flocks (*Gallus gallus*) on production flocks [5] reached only qualitative conclusions, owing to the lack of EU-wide harmonised data and to the shortcomings of models that did not consider harmonised *Salmonella* control practices implemented at the time in the EU. Nevertheless, the conclusions of the assessment provided a useful indication of the relevance of *S. enterica* and *S.* Enteritidis in the vertical transmission of *Salmonella* from breeding to production stock. Estimating that reducing the already low target for *Salmonella* in breeding flocks would have a relative small impact on prevalence in production flocks triggered reconsiderations for setting a new target in that type of poultry.

Assessing the public health impact of setting a new target in laying hen flocks [6] was supported with quantitative estimates based on the modelling of *S.* Enteritidis in table eggs [11]. Adequate data were available from only two MSs, which impacted on the overall confidence and extrapolation of the results to the whole EU. Moreover, post-farm factors and other *Salmonella* serovars were not considered. The lack of data hampered the quantitative estimation of the significance of other food-borne transmission routes of *Salmonella* to humans from the laying hen reservoir, which included consumption of egg products and meat from spent hens. Thus, the quantitative estimates were highly uncertain but, still, the assessment provided useful views on the relative benefit for public health of the transitional target and the proposed final target, compared to the reported *Salmonella* prevalence in laying hen flocks in the EU MSs in 2008. 

The assessment of the impact of setting new targets in broiler flocks of *Gallus gallus* [7] and turkey production flocks [8] was supported by the work of external contractors [15,18], who developed the mathematical models to underpin the quantitative assessments. Data were used from 22 and 25 MSs respectively. For both the human and animal-food source data, it was concluded that the lack of harmonised monitoring of human salmonellosis in the EU, as well as the different levels of serovar detail, were the main factors that contributed to the uncertainty of the model results, apart from the statistical uncertainties. Moreover, the model only included turkeys, pigs, laying hens and broilers as putative reservoirs. Some *Salmonella* reservoirs (e.g., cattle, other poultry, companion animals, reptiles) were not included in the model due to lack of data. It is therefore likely that the contribution of the human salmonellosis cases allocated to the animal reservoirs included in the model have been overestimated. In particular, *S.* Typhimurium was attributed mainly to the pig reservoir whereas it is likely that a considerable number of cases may actually be related to the cattle reservoir. 

The scientific opinions of the BIOHAZ Panel outlined in this paper are the result of the interaction of several actors. First of all, the four *ad hoc* working groups, consisting of experts from the BIOHAZ Panel and of additional external experts, who drafted the four scientific opinions for consideration by the BIOHAZ Panel. These *ad hoc* working groups were supported in some cases by the EFSA Unit on Scientific Assessment Support or by external contractors [15,18], who developed the mathematical models that provided quantitative estimates. And finally, the BIOHAZ Panel itself, who revised, finalised and adopted the scientific opinions. In the context of the EU food safety risk analysis framework, the scientific opinions were then published and delivered by EFSA to the European Commission, who then considered possible risk management measures.

The four assessments carried out by EFSA have enabled risk managers to set permanent EU targets to protect the public health of European consumers. More specifically, the transitional EU-wide *Salmonella* targets in flocks of breeding hens of *Gallus gallus* (Reg. (EC) No. 200/2010), laying hens of *Gallus gallus* Reg. (EC) No. 517/2011), broilers (Reg. (EC) No. 200/2012) and breeding and fattening turkeys (Reg. (EC) No. 1190/2012) have all been confirmed. The number of human salmonellosis cases reported in the EU decreased in the period from 2008–2011 from 153,852 cases to 97,897 cases. It is assumed that the observed reduction in salmonellosis cases is mainly as a result of the successful *Salmonella* control programmes in poultry populations. Most MSs met their *Salmonella* reduction targets for poultry in 2011 and *Salmonella* is declining in these animal populations [19]. It should be noted that microbiological criteria are also in place, in accordance to Regulation (EC) No. 2073/2005. According to these criteria, *Salmonella* must be absent in samples of several food categories, including minced meat, meat preparations and meat products from poultry origin.

In general, the EFSA BIOHAZ Panel acknowledged that these *Salmonella*-poultry related assessments should be reviewed over time. These revisions would benefit from additional yearly data from the harmonised monitoring of *Salmonella* in the different poultry populations and, thus, potentially result in the provision of better and more robust data-based assessments.
